# Catarrhal Inflammation of the Antrum

**Published:** 1866-03

**Authors:** Isaac Douglass

**Affiliations:** Romeo, Mich.


### CATARRHAL INFLAMMATION OF THE ANTRUM.

BY DR. ISAAC DOUGLASS, ROMEO, MICH.

[Read before the Michigan Dental Association.]

THE pituitary membrane of the antrum, or maxillary sinus, may be considered as a nearly closed, sack-like appendage, or continuation, of the schneiderian membrane. When in a healthy condition, it is constantly lubricated by a slightly glutinous fluid, which is transparent, inodorous, but slightly acid. Inflammation changes this secretion, both in quantity and quality, first lessening, and then increasing the quantity; as it is increased by the increasing inflammation, it becomes more or less vitiated, according to the degree of the inflammatory action.

DIAGNOSIS, SYMPTOMS.—Sensation of chilliness, on exposure to air and on moving, inclination to get near the fire, cold feet and hands, or else burning in the feet, heat and heaviness about the head during the disease; with frequently a burning sensation in the external ear, on the side affected, a full or swollen sensation of the cheek (or cheeks, as the case may be), without any perceptible change (that is to vision), except a slight fullness and increased redness of the eye of the affected side. A fixed, heavy, pressed sensation in the sinus, at first slight, but gradually increasing in severity, until it becomes, or is attended with, a deep seated, heavy, aching pain. The teeth below the sinus seem elongated, tender on shutting the teeth together, but not to lateral pressure, yet a constant inclination to shut them. Soreness of the facial muscles, but principally the muscles of mastication. Usually the first noticeable change in the secretions is a thickened consistency and tenacity, then it takes on a pale yellow tinge, gradually deepening to lemon color, becoming muco-purulent, but still retaining its glutinons character; or it may change to an orange color (but in that case it is tough, ropy, and difficult of detachment, so that if it flows back into the throat, and is coughed up, it may be drawn from the mouth with the fingers, even giving pain in the antrum as it is thus detached by being drawn through this circuitous rout). As the disease progresses, the secretion becomes less ropy, tinged with, or considerably mixed, with blood, or degenerates into a muco-purulent, greenish, offensive matter, disgusting to the patient and all around him.

Under either of the above conditions the discharge may be so acrid as to irritate the schneiderian membrane and the fauces to such an extent as to be even more difficult to bear than all the suffering in the region of the antrum. The pulse varies very much, sometimes full, bounding, with an increase of 10 or 20 beats, or it may be small, wiry, with an increase of from 5 to 30 beats per minute, with a firm quick stroke; tongue coated white or yellow.

Causes.—Exposure to cold or wet when unduly heated. Acids generated in the stomach. Imperfect digestion and assimilation of food, and consequent bad condition of the blood. Scrofulous taint in the system, or hereditary predisposition to catarrhal affections. Often repeated violent efforts to clear the nasal passages by blowing the nose during common catarrh.

Patohlogy. — This disease consists in a specific irritation of the mucous surfaces of the sinus. It is believed that the morbific influences which originate catarrhal inflammation (whether in this cavity, the nostrils, frontal sinuses, eyes, posterior nares, fauces, throat, pharynx, æsophagus, glottis, trachea or bronchial tubes) or all together affect, primarily, the organic nerves which supply the surfaces principally disordered, and, through them, the system generally. Owing to this change in the condition of the diseased texture, the functions of secretion and circulation in the part are specially deranged. The chief modifications of the disease arise from the degree in which the constitutional actions are disturbed, and grade of irritation produced in the capillaries of the part.

Prognosis.—Catarrh of the cavity is generally a mild ailment, attended with little or no danger, under a judicious course of treatment. But in aged people, or in scrofulous subjects, who are much debilitated, it may prove unfavorable. Obstinate and difficult cases are characterized by continued irritation, abundant muco-puriform secretion, with closing of the duct; or from the acrid secretion constantly flowing back into the throat, it may communicate the diseased condition of the posterior nares, fauces, throat, pharynx, œsophagus, and soon through the alimentary canal. Or, on the other hand, it may be communicated to the glottis, trachea and bronchial tubes, and thus terminate in chronic bronchitis.

Treatment.—Aconite drop doses of 3d potency. Almost always in the commencement of the treatment, even if the fever is very slight, also when on an increase of the cold, the discharge is suppressed, it is followed by headache, or increased fever and chilliness.

Podophylin; 2nd potency.—When there is disordered stomache, headache with heaviness, burning sensation in the scalp and external ears, heavy coating on the tongue, discharge orange color.

ARSENICUM; 3d potency one grain. — When there is not much fever, the patient wants to drink often, but little at a time, feels very weak, and is easily agitated, discharge acrid and corrosive, with burning and soreness or sensitiveness of the posterior nares; exercise and warmth are agreeable, and yet exposure does not aggravate the disease.

NUX-VOMICA; 3d potency drop doses. — When arsenicum fails to relieve, or the mouth is dry and parched, without much thirst, constipation and great heat of the head and face, fever and chills alternate in the evening.

PULSATILLA; 2d dilution, drop doses. — Loss of appetite, and small discharge, thick yellow (lemon color), or greenish and offensive, or streaked with blood.

HYDRASTIS CANADENSIS — Discharge ropy, tough, difficult to detach, excessive accumulation in the throat. This remedy may be alternated with podophylin, with good effect, when the discharge is orange colored and tastes like raw eggs. Dose of hydrastis tincture, in drop doses, of 3d dilution.

An occasional dose of sulphur, drop dose, once in 2 or 3 days, 3d potency, often does much to eradicate the disease, where it assumes a chronic form.

DIET, &c.—It is highly important that the patient have a light diet, gruels toast water, or dry toast, scalded milk, farina, corn starch, &c., &c. Hot foot baths will do much towards arresting this disease in its early stages, particularly if the feet are inclined to be cold; but in order to get much benefit from them, they should be continued for at least 20 or 30 minutes, adding a little hot water as often as can be borne.

Doses of any of the above remedies, except sulphur, may be repeated 2 or 3 hours apart.

